# The Little Things in Obstetrics

**Published:** 1917-05

**Authors:** W. G. Harris

**Affiliations:** Plano, Texas


### The Little Things in Obstetrics.

BY DR. W. G. HARRIS, PLANO, TEXAS.

The little thing in obstetrics has long been neglected in the interest in the human race. Is it not more the physician's true calling to prevent, than to treat that which might have been prevented? If so, then we must continually endeavor to educate the public—iterate and reiterate such tenets as "like begets like." If, as has been said, "the child is the father of man," I say that the fetus is rather the father of the man. This being the case, then we have an endless chain, and as a chain is no stronger than its weakest link, then should we not seek for the weakest link and endeavor to teach the people how to prevent such a great mortality in the little thing? Why this great mortality? The answer comes back in one word—ignorance. If any modern business industry had such a great per cent of loss as there is in this little factor, it would put a high-priced expert on the job; then after getting its bearings, it would say that the inefficient and dead weights not only should, but must be eliminated. Do you grant that it is incumbent upon the people to maintain our eleemosynary institutions? If you do, then you will have to admit that those who keep up these institutions should be the sole arbiters in preventing the making of the inmates of these institutions. Shall we legislate against this evil? No, not now. We must first educate the people. Be a statesman and tell the people what is best for them—not a politician fawning to a fickle following. Then and only then will legislation follow easily as a natural consequence. We should teach by precept and make examples of degenerates, whenever permitted by operating upon them, not to emasculate but so that they cannot propagate. There are too many born just to consume the corn, as Horace says. Every young woman who intends to marry or have offspring should be taught, in a school for that purpose, how to care for herself and the offspring which she may bear, and being so taught, she might decide that matrimony and maternity were not best for her, instead of bringing into existence a puny degenerate, dependent upon others for support. The Better Baby Shows will have a tendency to do good in that they will stimulate interest in better babies and the question will naturally follow: How are we to have better babies? The answer is easy: By having better parents. But how are we to have better parents? By having better babies. Now you see that we are still within the endless chain, and the only way to have better babies and improve the race is to educate as far as possible and then to eliminate the unfit as parents. Who are the unfit? The degenerates, the imbecile, the confirmed epileptic, the desperately criminal, the hopeless tubercular, the syphilitic who will not be treated and cured, and the persistent drug and liquor addict. Do you not know that a large per cent of these care more for their sexual pleasure than they do for their offspring? Why should not these be sterilized in the interest of the human race, rather than have the race burdened with a load which is ever increasing? Is it sentimentality? Then you too should be educated. Have you not known of marriages where parents have given their daughters willingly to men, on account of their social and financial standing, who were not worthy of the name of men? And still these same parents would not have permitted males of a different species, which graded no higher than these sons-in-law, to head their herds of scrub cattle. And what shall I say of the woman who marries for affluence and ease? Does she not virtually sell herself? Is there any difference in selling one's self for a lifetime, a year, or a day? I fail to see the difference, only in the price paid. Is the mortality of the little thing to be wondered at under the present social conditions with mishaps, misfits and such human accidents?

